# ALDH1A1 promotes PARP inhibitor resistance by enhancing retinoic acid receptor-mediated DNA polymerase θ expression

**DOI:** 10.1038/s41698-023-00411-x

**Published:** 2023-07-10

**Authors:** Kousalya Lavudi, Ananya Banerjee, Na Li, Yajing Yang, Shurui Cai, Xuetao Bai, Xiaoli Zhang, Aidan Li, Elsa Wani, Shyh-Ming Yang, Junran Zhang, Ganesha Rai, Floor Backes, Srinivas Patnaik, Peixuan Guo, Qi-En Wang

**Affiliations:** 1 Department of Radiation Oncology, College of Medicine, The Ohio State University, Columbus, OH 43210 USA; 2 Comprehensive Cancer Center, The Ohio State University, Columbus, OH 43210 USA; 3 Department of Biomedical Informatics, College of Medicine, The Ohio State University, Columbus, OH 43210 USA; 4 Washington University in St. Louis, St. Louis, MO 63130 USA; 5 Division of Preclinical Innovation, National Center for Advancing Translational Sciences, National Institutes of Health, Rockville, MD 20850 USA; 6 Department of Obstetrics & Gynecology, Division of Gynecologic Oncology, College of Medicine, The Ohio State University, Columbus, OH 43210 USA; 7 School of Biotechnology, Kalinga Institute of Industrial Technology, Bhubaneswar, Odisha 751024 India; 8 Center for RNA Nanobiotechnology and Nanomedicine, Division of Pharmaceutics and Pharmacology, College of Pharmacy, The Ohio State University, Columbus, OH 43210 USA

**Keywords:** Cancer therapeutic resistance, Ovarian cancer

## Abstract

Poly (ADP-ribose) Polymerase (PARP) inhibitors (PARPi) have been approved for both frontline and recurrent setting in ovarian cancer with homologous recombination (HR) repair deficiency. However, more than 40% of BRCA1/2-mutated ovarian cancer lack the initial response to PARPi treatment, and the majority of those that initially respond eventually develop resistance. Our previous study has demonstrated that increased expression of aldehyde dehydrogenase 1A1 (ALDH1A1) contributes to PARPi resistance in BRCA2-mutated ovarian cancer cells by enhancing microhomology-mediated end joining (MMEJ) but the mechanism remains unknown. Here, we find that ALDH1A1 enhances the expression of DNA polymerase θ (Polθ, encoded by the *POLQ* gene) in ovarian cancer cells. Furthermore, we demonstrate that the retinoic acid (RA) pathway is involved in the transcription activation of the *POLQ* gene. The RA receptor (RAR) can bind to the retinoic acid response element (RARE) located in the promoter of the *POLQ* gene, promoting transcription activation-related histone modification in the presence of RA. Given that ALDH1A1 catalyzes the biosynthesis of RA, we conclude that ALDH1A1 promotes *POLQ* expression via the activation of the RA signaling pathway. Finally, using a clinically-relevant patient-derived organoid (PDO) model, we find that ALDH1A1 inhibition by the pharmacological inhibitor NCT-505 in combination with the PARP inhibitor olaparib synergistically reduce the cell viability of PDOs carrying BRCA1/2 mutation and positive ALDH1A1 expression. In summary, our study elucidates a new mechanism contributing to PARPi resistance in HR-deficient ovarian cancer and shows the therapeutic potential of combining PARPi and ALDH1A1 inhibition in treating these patients.

## Introduction

Poly (ADP-ribose) polymerase (PARP) inhibitors (PARPi) are a novel class of anti-cancer agents that have shown efficacy for tumors with certain DNA repair gene mutations that lead to homologous recombination (HR) deficiency (HRD). Several PARPi have been approved for the treatment of BRCA-mutated ovarian cancer, breast cancer, pancreatic cancer, and prostate cancer. PARPi binds to the catalytic domain of PARP1/2, inhibiting their enzymatic activity to repair DNA single-strand breaks (SSBs), and trapping PARP1/2 on DNA as well^1^. The unrepaired DNA gaps and the PARP/DNA complexes are further converted to more deleterious DNA double-strand breaks (DSBs), mainly through collapse of stalled replication forks during DNA replication. Error-free HR is primarily responsible for the repair of DNA DSBs. Since BRCA-mutated cells are unable to perform HR to repair DSBs, these cells are more sensitive to PARPi through the mechanism of synthetic lethality^2,3^.

Although many patients with BRCA deficiency respond to PARPi at the beginning, resistance can develop, and tumors can progress. The mechanisms behind PARPi resistance have been considered multi-factorial, including increased DSB repair, increased drug efflux, decreased PARP trapping, and stabilization of stalled forks^4^. Given that HR is the predominant DNA repair pathway to repair DSBs, the full or partial restoration of HR via BRCA1/2 secondary reverse mutation, or epigenetic modification of multiple DSB repair genes, is considered the most common acquired mechanism of PARPi resistance in HR-deficient tumors^4^. Besides HR, DSBs can also be repaired by error-prone repair pathways such as classic non-homologous end joining (c-NHEJ), and alternative end joining (Alt-EJ, also called microhomology-mediated end joining, MMEJ)^5^. In c-NHEJ, the DSB is repaired by blunt end ligation independently of sequence homology, requiring many factors such as Ku70/80, DNA-PKcs, and DNA ligase IV. In contrast, MMEJ needs end resection of DSBs, and repairs DSBs by using 5 to 25-base pair micro-homologous sequences to align the broken strands before joining. This repair pathway requires DNA polymerase θ (Polθ, coded by the *POLQ* gene)^5^. These error-prone repair pathways can compensate for the loss of HR and play a major role in DSB repair in HR-deficient cells^6^. However, c-NHEJ was found to contribute to the toxicity of PARPi in HR-deficient cells, probably via NHEJ-mediated genomic instability^7^, while MMEJ can increase the cell viability in HR-deficient cells after PARPi treatment^6^, indicating that enhanced MMEJ can be a mechanism underlying PARPi resistance in HR-deficient cancer cells^8^. Our previous study has demonstrated that PARPi treatment can induce the expression of aldehyde dehydrogenase 1A1 (ALDH1A1), which further enhances MMEJ to facilitate the repair of DSBs. However, the mechanism underlying ALDH1A1-mediated MMEJ is still unclear.

Aldehyde dehydrogenases (ALDH) are a family of enzymes that can catalyze the conversion of both endogenous and exogenous aldehydes to lesser toxic carboxylic acids^9^. Nineteen human ALDH isoforms have been identified, which participate in a wide variety of biological processes in cells by employing their diverse catalytic functions, including promoting retinoic acid (RA) biosynthesis^10^. ALDH, particularly the ALDH1 subfamily, participates in the conversion of retinal to RA, which binds to the nuclear receptors retinoic acid receptor (RAR) and retinoid X receptors (RXR) located in the retinoic acid response element (RARE) in the promoter region, relaxing local chromatin structure, and promoting transcription of the downstream target genes^11^.

In this study, we provide evidence showing that ALDH1A1 enhances MMEJ and PARPi resistance via promoting the expression of Polθ. Mechanistically, ALDH1A1 facilitates the production of RA, which binds to the RAR to activate the RAR signaling pathway. We also identified a putative RARE in the promoter region of *POLQ* and elucidated the regulatory mechanism of *POLQ*/Polθ expression by ALDH1A1. Finally, we demonstrated that a combination of olaparib with an ALDH1A1 inhibitor, NCT-505, can synergistically reduce the viability of patient-derived organoids (PDOs) generated from primary high-grade serous ovarian carcinoma (HGSOC) tumor tissues, which possess mutant BRCA1/2 and positive ALDH1A1 expression. The findings in this study demonstrated a new mechanism contributing to the development of PARPi resistance in HR-deficient ovarian cancer and showed the therapeutic potential of combining PARPi and ALDH1A1 inhibition in treating these patients.

## Results

### ALDH1A1 enhances Polθ expression

**Fig. 1:**
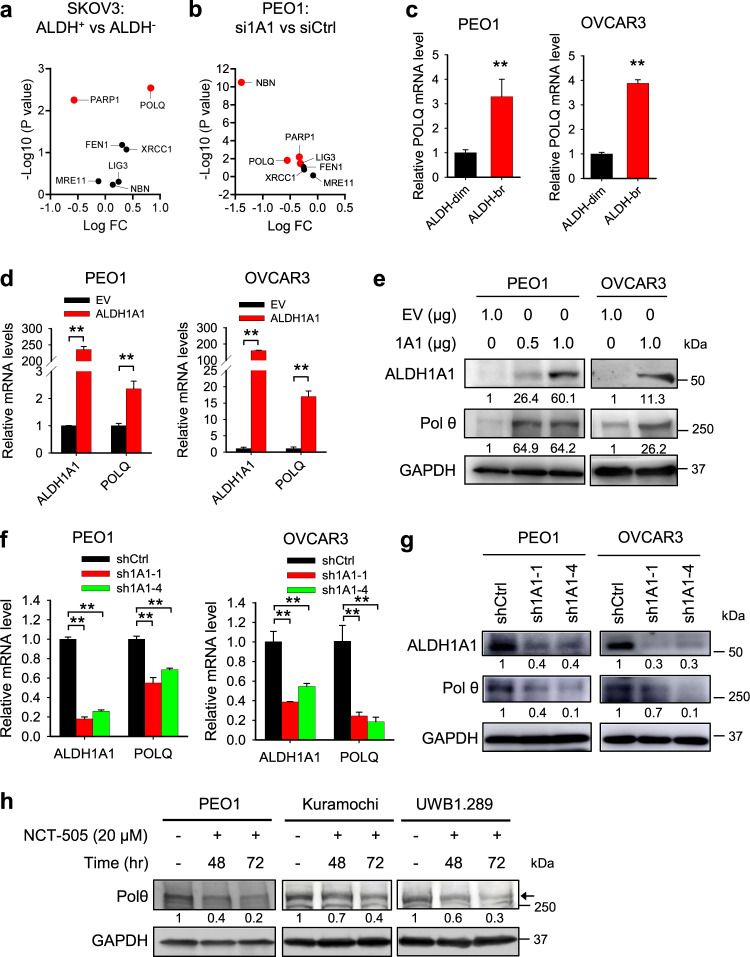
ALDH1A1 enhances Polθ expression. **a** Volcano plot depicting differentially expressed MMEJ-related genes in ALDH^+^ vs ALDH^-^ cells isolated from SKOV3 cells. **b** Volcano plot depicting differentially expressed MMEJ-related genes in siALDH1A1 transfected vs siControl transfected PEO1 cells. **c** The *POLQ* mRNA level is higher in ALDH-br cells than that in ALDH-dim cells isolated from PEO1 and OVCAR3 cells. **d** Overexpression of ALDH1A1 increased the mRNA level of *POLQ*. **e** Overexpression of ALDH1A1 increased the protein level of Polθ. **f** Knockdown of ALDH1A1 decreased the mRNA level of *POLQ*. **g** Knockdown of ALDH1A1 decreased the protein level of Polθ. **h** Inhibition of the activity of ALDH1A1 with NCT-505 reduced the expression of Polθ. Measurements were taken from distinct samples. *N* = 3, bar: s.d., **P < 0.01. The relative amounts of analyzed proteins were listed under the corresponding band. The arrow indicates the specific Polθ band.

We have previously demonstrated that ALDH1A1 enhances MMEJ in ovarian cancer cells^12^. However, the mechanism underlying ALDH1A1-mediated MMEJ regulation is still unknown. Polθ, encoded by the *POLQ* gene, plays a central role in MMEJ^6,13–15^. In addition, at least 6 other genes are required for MMEJ, e.g., *FEN1*, *Ligase III*, *MRE11*, *NBS1*, *PARP1* and *XRCC1*^16^. To determine whether the expression of these genes can be regulated by ALDH1A1, we first analyzed the microarray data in a publicly available dataset (GSE82304) that contains gene expression information in ALDH^+^ vs ALDH^-^ cells isolated from an ovarian cancer cell line SKOV3^17^. Among all seven MMEJ-related genes, only *POLQ* (LogFC: 0.829; *P* value: 0.003) exhibits a significantly enhanced expression in ALDH^+^ cells compared to ALDH^-^ cells (Fig. 1a and Supplementary Fig. 1A). We further analyzed gene expression in PEO1 cells transiently transfected with ALDH1A1 siRNA or control siRNA using RNA-seq. We found that only *POLQ* (LogFC: −0.5518; *P* value: 0.0154) and *NBN* (encoding NBS1, a component of the MRE11-RAD50-NBS1 complex, LogFC: −1.3886; *P* value: 3.19E-11) genes are significantly downregulated by ALDH1A1 siRNA (Fig. 1b and Supplementary Fig. 1A, B). Taken together, it appears that among these MMEJ-related genes, the *POLQ* gene is the only one that is highly expressed in ALDH^+^ cells, and can be downregulated by ALDH1A1 siRNA. Next, we sorted ALDH-dim and ALDH-bright (ALDH-br) cell subpopulations from two ovarian cancer cell lines PEO1 and OVCAR3, and validated the enhanced expression of *POLQ* at the mRNA level in ALDH-br cells compared to ALDH-dim cells using qRT-PCR (Fig. 1c).

To specifically validate the regulation of the POLQ expression by ALDH1A1, we overexpressed ALDH1A1 in PEO1 and OVCAR3 cells, and determined *POLQ*/Polθ expression. We found that ALDH1A1 overexpression can enhance *POLQ*/Polθ expression at both mRNA and protein levels in these two cell lines (Fig. 1d, e). The upregulation of *POLQ*/Polθ by ALDH1A1 is further confirmed in another ovarian cancer cell line Kuramochi (Supplementary Fig. 1C, D). Furthermore, we knocked down the ALDH1A1 expression using ALDH1A1 shRNA in PEO1, OVCAR3, and Kuramochi cells, and found that *POLQ*/Polθ expression is downregulated by ALDH1A1 shRNA as well (Fig. 1f, g and Supplementary Fig. 1E, F). In addition, treatment of PEO1, Kuramochi and UWB1.289 cells with a specific ALDH1A1 inhibitor, NCT-505^18^, also reduced the expression of Polθ (Fig. 1h), further confirming the positive regulation of *POLQ*/Polθ by ALDH1A1. Taken together, these data indicate that ALDH1A1 is able to upregulate the expression of Polθ, which could be the mechanism by which ALDH1A1 enhances MMEJ.

### ALDH1A1 enhances DNA repair and PARPi resistance via upregulating Polθ

**Fig. 2:**
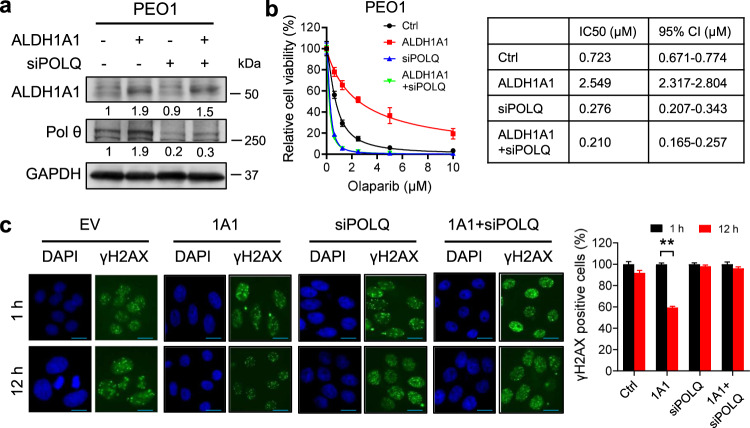
ALDH1A1 enhances DNA repair and PARPi resistance via upregulating Polθ. PEO1 cells were transfected with ALDH1A1 expression plasmids or/and siPOLQ for 48 h. The expression of ALDH1A1 and Polθ was determined using immunoblotting (**a**). The relative amounts of ALDH1A1 and Polθ were listed under the corresponding band. These cells were treated with olaparib at various doses for 7 days, the cell viability and IC50 were determined using the methylene blue assay (**b**). Cells were treated with olaparib (10 μM) for 1 h, and further cultured in the drug-free medium for 1 and 12 h. γH2AX foci were visualized using immunofluorescence. The percentage of γH2AX positive cells (>5 foci/cell) was calculated and normalized to 1 h time point (**c**). Measurements were taken from distinct samples. *N* = 5, bar: s.d., **P < 0.01. Scale bar: 20 μm.

Overexpression of ALDH1A1 is able to enhance DNA repair and render ovarian cancer cells resistant to olaparib treatment^12^. To determine whether ALDH1A1-mediated upregulation of Polθ plays a critical role in this process, we overexpressed ALDH1A1 in PEO1 cells, meanwhile, we blocked *POLQ***/**Polθ expression by simultaneously transfecting cells with *POLQ* siRNA (Fig. 2a), and then treated these cells with olaparib. As shown in Fig. 2b, overexpression of ALDH1A1 significantly reduced the sensitivity of PEO1 cells to olaparib, with the IC50 increased from 0.723 μM to 2.549 μM. In contrast, transfection with siPOLQ sensitized PEO1 cells to olaparib. More importantly, simultaneous transfection with ALDH1A1 and siPOLQ antagonized ALDH1A1-induced olaparib resistance (Fig. 2b). These findings were also validated in another BRCA2-mutated ovarian cancer cell line Kuramochi (Supplementary Fig. 2A, B), indicating that Polθ plays a central role in ALDH1A1-mediated olaparib resistance. We further examined the role of Polθ in ALDH1A1-mediated DNA repair. HR-deficient PEO1 and Kuramochi cells were transfected with ALDH1A1 expression plasmids or/and siPOLQ and treated with olaparib to induce DNA damage. γH2AX foci in these cells were analyzed at different time points to evaluate the DNA repair capability. As expected, overexpression of ALDH1A1 enhanced the repair of olaparib-induced DNA damage in both cell lines, reflected by faster disappearance of γH2AX foci in these cells after 12 h of olaparib treatment, while simultaneous transfection with *POLQ* siRNA blocked ALDH1A1 overexpression-induced disappearance of γH2AX foci (Fig. 2c and Supplementary Fig. 2C), indicating that Polθ plays an important role in ALDH1A1-induced enhancement of DNA repair after olaparib treatment. Taken together, these data demonstrate that ALDH1A1 enhances DNA repair and PARPi resistance via upregulating Polθ.

### ALDH1A1 promotes the *POLQ*/Polθ expression by increasing the production of RA

**Fig. 3:**
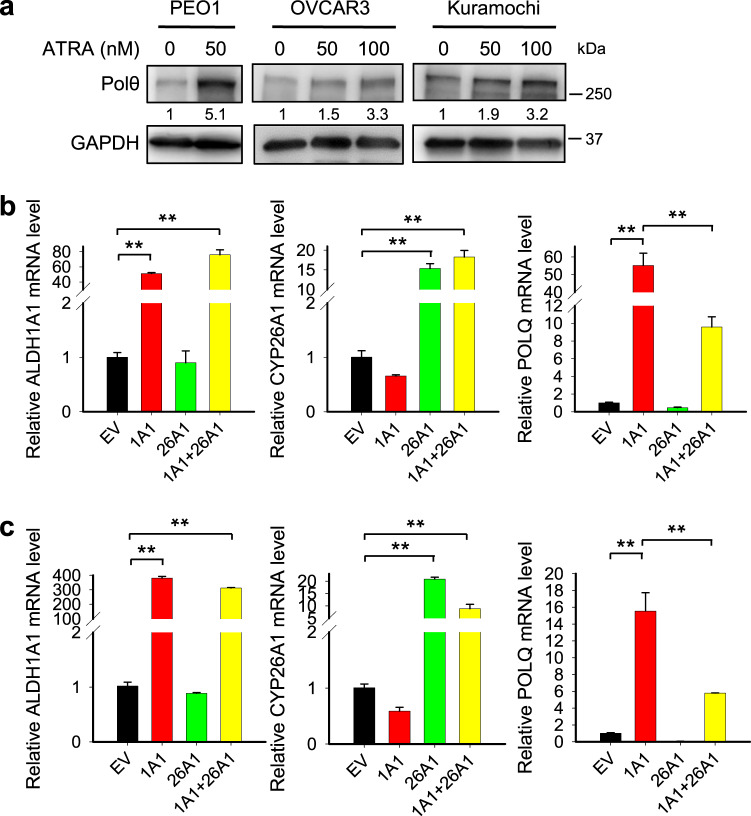
ALDH1A1 promotes the *POLQ*/Polθ expression by increasing the production of RA. **a** PEO1, OVCAR3, and Kuramochi cells were treated with ATRA for 48 h, immunoblotting was conducted to determine the expression of Polθ. The relative amounts of Polθ were listed under the corresponding band. **b**, **c** PEO1 (**b**) and Kuramochi (**c**) cells were transfected with ALDH1A1 or/and CYP26A1 for 48 h, qRT-PCR was conducted to determine the mRNA level of *ALDH1A1, CYP26A1*, and *POLQ*. Measurements were taken from distinct samples. *N* = 3, bar: s.d., **P < 0.01.

One of the major functions of ALDH1A1 is to catalyze the conversion of retinal to RA, which can further activate the RA signaling pathway to regulate gene expression^19,20^. To determine whether ALDH1A1 upregulates the *POLQ*/Polθ expression via RA, we first treated PEO1, OVCAR3 and Kuramochi cells with All-*trans*-Retinoic Acid (ATRA), the principal activator of RA signaling, and examined the protein level of Polθ in treated cells. As shown in Fig. 3a, ATRA treatment can significantly increase the expression of Polθ. We then overexpressed ALDH1A1 in PEO1 and Kuramochi cells, while co-transfecting cells with CYP26A1, an enzyme to inactivate RA^21,22^. The overexpression of ALDH1A1 and CYP26A1 in the correspondingly transfected cells were confirmed using qRT-PCR (Fig. 3b, c). Same as the results shown in Fig. 1, ALDH1A1 overexpression increased the mRNA level of *POLQ*, while simultaneous overexpression of CYP26A1 inhibited such effect (Fig. 3b, c). Taken together, these results indicate that ALDH1A1 promotes the *POLQ*/Polθ expression by increasing the production of RA.

### *POLQ*/Polθ expression is regulated by RAR signaling

**Fig. 4:**
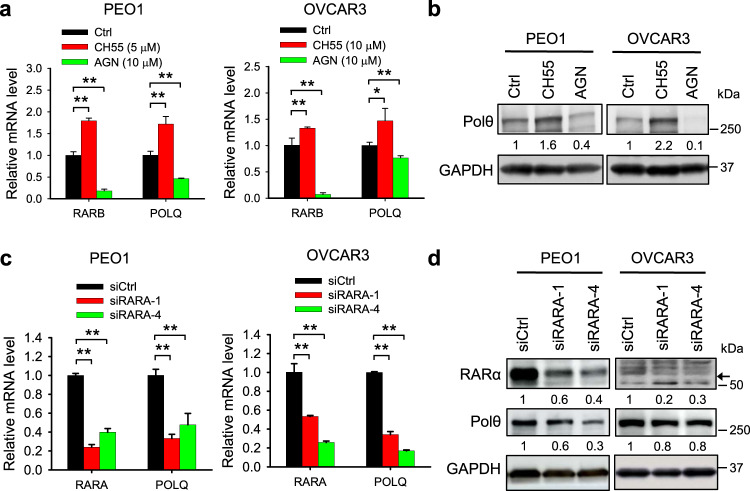
*POLQ*/Polθ expression is regulated by RAR signaling. **a, b** PEO1 and OVCAR3 cells were treated with RAR agonist CH55 or RAR antagonist AGN 193109 for 48 h, qRT-PCR and immunoblotting were conducted to determine the mRNA (**a**) and protein (**b**) levels of *POLQ*/Polθ, respectively. The mRNA level of *RARB* was also determined to validate the effect of RAR agonist and antagonist on the RAR signaling. **c**, **d** PEO1 and OVCAR3 cells were transfected with two *RARA* siRNA for 48 h, qRT-PCR and immunoblotting were conducted to determine the mRNA (**c**) and protein (**d**) levels of *RARA*/RARα and *POLQ*/Polθ, respectively. Measurements were taken from distinct samples. *N* = 3, bar: s.d., **P < 0.01. The relative amounts of analyzed proteins were listed under the corresponding band. The arrow indicates the specific RARα band.

Given that RA can activate the RA signaling pathway via binding to the RAR, we reasoned that the ATRA treatment-induced *POLQ*/Polθ expression could be mediated by RAR. To test this hypothesis, we first activated or inhibited the RAR using its selective agonist CH55 or antagonist AGN 193109, respectively, and examined the expression of *POLQ*/Polθ in a panel of ovarian cancer cell lines. The expression level of RARB, a direct downstream gene of RAR, was also determined to validate the effect of the RAR agonist and antagonist. We found that CH55 significantly increased while AGN 193109 significantly decreased the mRNA level of both *RARB* and *POLQ* in all tested cells (Fig. 4a and Supplementary Fig. 3A). The changes in Polθ was also confirmed using immunoblotting (Fig. 4b and Supplementary Fig. 3B). Next, we knocked down the expression of RARα using two individual siRNA that target distinct regions of *RARA* and determined the changes in *POLQ*/Polθ expression. Both qRT-PCR and immunoblotting analyses showed reduced *RARA* and *POLQ*/Polθ at mRNA and protein levels (Fig. 4c, d and Supplementary Fig. 3C, D). All these results indicate that the expression of *POLQ*/Polθ can be regulated by the RAR signaling pathway.

### *POLQ* is a direct downstream gene of RAR signaling

**Fig. 5:**
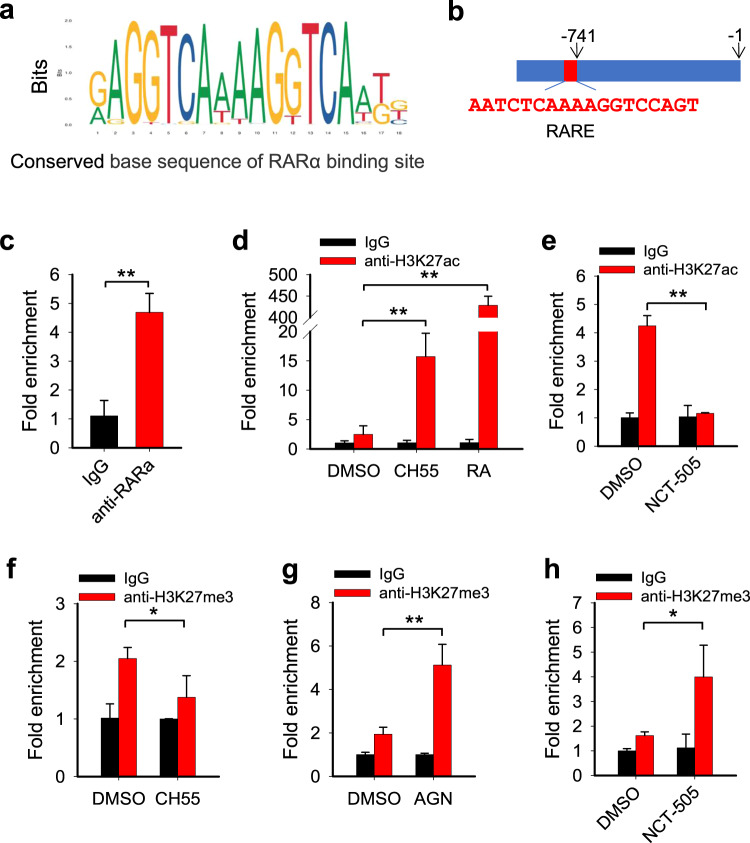
*POLQ* is a direct downstream gene of RAR signaling. **a** The conserved base sequence of RARα binding site was identified using JASPAR. **b** The putative RARE was identified in the promoter region of the *POLQ* gene. **c** The ChIP assay with the anti-RARα antibody and normal IgG was conducted to analyze the binding of RARα to the promoter region of the *POLQ* gene. **d**, **e** The ChIP assay with the anti-H3K27ac antibody was conducted to determine the effect of RAR agonist CH55, RA (**d**), and ALDH1A1 inhibitor NCT-505 (**e**) on the histone H3K27 acetylation around the RARE region of the *POLQ* gene. **f**–**h** The ChIP assay with the anti-H3K27me3 antibody was conducted to determine the effect of CH55 (**f**), RAR antagonist AGN 193109 (**g**), and NCT-505 (**h**) on the histone H3K27 methylation around the RARE region of the *POLQ* gene. Measurements were taken from distinct samples. *N* = 3, bar: s.d., **P < 0.01.

It is well known that RA binds to RAR or RXR, which binds to RAREs or retinoid X response elements (RXREs), the well-defined DNA sequences in promoters or enhancers, to regulate gene expression (Fig. 5a)^23^. We indeed identified a RARα binding site in the *POLQ* gene promoter region using JASPAR (Fig. 5b). We then performed the Chromatin immunoprecipitation (ChIP) assay using the anti-RARα antibody to determine whether RARα can bind to this region in ovarian cancer cells. The ChIP analysis shows that RARα is indeed enriched in the *POLQ* gene promoter region containing the putative RARE sequence (Fig. 5c). Given that the binding of RA to RAR can release the transcription repressive Polycomb repressive complex 2 (PRC2) and histone deacetylase (HDAC), and recruit the transcription permissive Trithorax and histone acetyltransferase (HAT)^11^, results in a decrease in H3K27 methylation (H3K27me3) and an increase in H3K27 acetylation (H3K27ac), thereby activating the transcription of the target genes^24^, we analyzed the effect of RAR activation or inhibition and ALDH1A1 inhibition on the enrichment of these modified histones in the *POLQ* gene promoter region. The ChIP analyses demonstrate that CH55 and RA treatment can significantly enhance, while NCT-505 treatment can significantly reduce the enrichment of transcriptional activation mark H3K27ac (Fig. 5d, e). In contrast, CH55 reduced, while AGN 193109 and NCT-505 treatment enhanced the enrichment of transcriptional repressive H3K27me3 (Fig. 5f–h), in the *POLQ* gene promoter. These results further indicate that RA-activated RARα can be enriched in the *POLQ* promoter and facilitate *POLQ* transcription by promoting the histone H3 acetylation and inhibiting the histone H3 methylation at lysine 27.

### Inhibition of ALDH1A1 synergistically enhances the cellular toxicity of olaparib in primary HGSOC tumor tissues

**Fig. 6:**
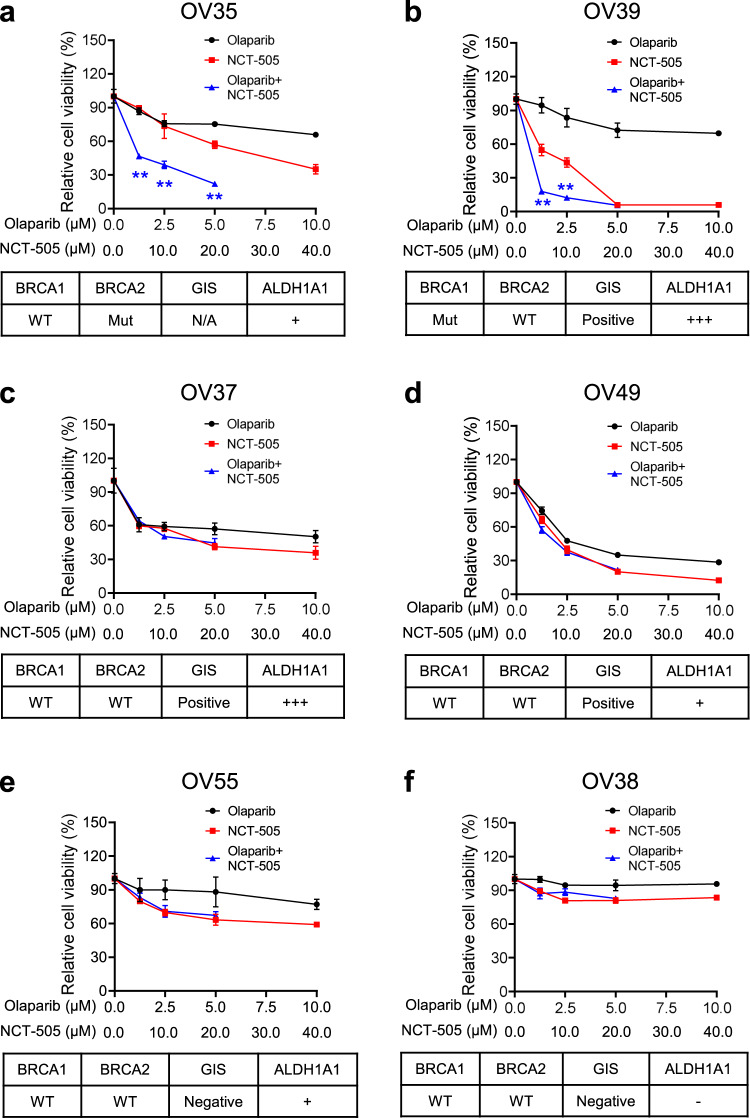
ALDH1A1 inhibitor synergistically enhances the cytotoxicity of olaparib in PDOs generated from primary HGSOC tumor tissues with BRCA1/2 mutation and positive ALDH1A1 expression. PDOs were generated from primary HGSOC tumor tissues, treated with olaparib or/and NCT-505 for 7 days. The CellTiter-Glo 3D cell viability assay was used to determine the cell viability after treatment. The BRCA1/2 mutation status and the GIS of these tumor tissues were obtained and the ALDH1A1 expression status was determined using IHC. **a** BRCA2-mut, ALDH1A1-positive. **b** BRCA1-mut, ALDH1A1-positive. **c**, **d** BRCA1/2-WT, GIS-positive, ALDH1A1-positive. **e** BRCA1/2-WT, GIS-negative, ALDH1A1-positive. **f** BRCA1/2-WT, GIS-negative, ALDH1A1-negative. Linear mixed-effect models were used to analyze the synergistic effect of olaparib and NCT-505 on the cell viability. Measurements were taken from distinct samples. *N* = 4, bar: s.d., **P < 0.01.

Our previous study has shown that a specific selective ALDH1A1 inhibitor NCT-501 is able to halt the growth of xenografts derived from an EOC cell line with low DDB2 expression^25^. We have also shown that NCT-501 can sensitize *BRCA2* mutated EOC cell lines to olaparib treatment^12^. To improve the bioavailability and efficacy, a second generation of ALDH1A1 inhibitor, NCT-505, was discovered, which exhibits high enzymatic potency and cellular activity in various cancer cell lines with a high selectivity in inhibiting ALDH1A1 over other ALDH isozymes^18^. To test the effect of NCT-505 on PARPi sensitivity in primary tumor cells, we collected a panel of freshly removed HGSOC tumor tissues and generated Patient-derived organoids (PDOs) according to a published protocol^26^. The tumor histopathology was confirmed by H&E staining; ALDH1A1 expression in these tissues was assessed using IHC (Supplementary Fig. 4). PDOs were treated with olaparib and NCT-505, separately or in combination, at various doses for 7 days. Cell viability analyses showed that NCT-505 and olaparib can synergistically reduce tumor cell viability in OV35 (Fig. 6a) and OV39 (Fig. 6b) HGSOC tumor organoids, which possess BRCA2 and BRCA1 mutation, respectively, as well as positive ALDH1A1 expression. In contrast, no synergy was observed in OV37 (Fig. 6c) and OV49 (Fig. 6d) PDOs, although these tissues possess positive GIS and positive ALDH1A1, but carry wild type (WT) BRCA1/2. Similarly, no synergy was observed in the OV55 PDOs (Fig. 6e), which possess WT BRCA1/2 and negative GIS, although the ALDH1A1 expression is positive. As expected, ALDH1A1-negative PDOs (OV38, Fig. 6f) do not show synergy either. These results indicate that inhibition of ALDH1A1 can only enhance the efficacy of olaparib in the treatment of HGSOC with BRCA1/2 mutation and high ALDH1A1 expression, while not those with wild type BRCA, regardless of ALDH1A1 expression.

## Discussion

PARPi offer an advantageous treatment option for patients with BRCA1/2 mutant tumors by exploiting synthetic lethality, but as with many cancer treatments, resistance eventually occurs. Although multiple mechanisms have been found, enhanced DNA repair, particularly the partial restoration of HR, is believed to be the major contribution to the development of PARPi resistance^4^. However, DNA DSBs are not only repaired by HR, the activation of other DSB repair pathways, e.g., MMEJ, also plays a critical role in resolving DSBs, particularly in HR-deficient cells^5,6,27^. Our previous study has revealed that PARPi treatment can induce the expression of ALDH1A1, which renders ovarian cancer cells resistant to PARPi by enhancing MMEJ^12^. In the current study, we further demonstrated that ALDH1A1 is able to elevate the expression of a critical player of MMEJ, Polθ, via activating the RA pathway, providing a novel mechanism underlying ALDH1A1-mediated PARPi resistance.

Like HR, MMEJ is initiated by 5’-3’ resection of DSB ends with the MRE11-RAD50-NBS1 (MRN) complex. Polθ then binds to long single-stranded DNA overhangs generated by end resection of DSBs and anneals sequences with 2–6 base pairs of microhomology to use them as primers for overhang extension. The DNA ends are then ligated by LIG3-XRCC1 or LIG1^28^. Therefore, augmentation of the MMEJ activity was recognized as one of the mechanisms underlying PARPi resistance in BRCA1/2-mutated cancer cells^6^, and targeting the MMEJ activity by inhibiting Polθ was considered a means to sensitize cancer cells to PARPi. Indeed, the antibiotic novobiocin, which was identified to function as an inhibitor of Polθ, can suppress the viability of HR-deficient tumor cells with acquired resistance to PARPi, and sensitized these cells to PARPi treatment^8^. Another Polθ inhibitor, ART558, also exhibited synthetic lethality in BRCA1- or BRCA2-mutant tumor cells and enhanced the cytotoxicity of PARPi^29^. However, Polθ is not merely involved in repair of DSBs, but also important for resolving stalled replication forks, as well as the repair of replication-associated DNA lesions and G4 quadruplex structure^30–33^, thereby helping maintain genomic stability in normal cells. Direct inhibition of Polθ may result in genomic instability and reduce the fitness and survival of normal cells. Therefore, identifying the mechanism underlying increased Polθ expression in PARPi-resistant cancer cells, and targeting this mechanism should be able to specifically inhibit the enhanced MMEJ by blocking the treatment-induced Polθ expression in these cells.

Elevated levels of ALDH1A1 is considered a marker of cancer stem cells, and is often associated with poor outcomes of various cancers^34,35^ and chemotherapy resistance^36,37^. Multiple mechanisms underlying ALDH1A1-mediated chemotherapy resistance have been revealed, such as detoxification of therapeutic drugs^38^, prevention of ROS-mediated apoptosis^36^, alteration of DNA repair networks^39^, and enhancement of nucleotide metabolic pathways^40^. In this study, we revealed for the first time that ALDH1A1 can upregulate the expression of the DNA repair factor Polθ. This upregulation occurs in both BRCA1/2-mutated and -WT cells, but only affects PARPi sensitivity in BRCA1/2-mutated cells, further supporting our previous findings that ALDH1A1 inhibitor can only be used to treat HR-deficient ovarian cancer cells in combination with PARPi^12^.

ALDH1A1 is the major isozyme in the ALDH enzyme family that catalyzes the production of RA^19^, and indirectly regulate the RA-mediated transcription of hundreds of genes^41^. ALDH1A1 preferentially convert all-trans retinal to all-trans retinoic acid (ATRA). ATRA is the signaling effector molecule and binds to retinoic acid receptor (RAR) or retinoid X receptor (RXR), which then binds to retinoic acid response elements (RAREs) or retinoid X response elements (RXREs), well-defined DNA sequences in promoters or enhancers, to regulate gene expression^23^. RA can regulate diverse cellular processes, including cell proliferation, tissue remodeling, and differentiation^42^. The ALDH1A1-RA axis plays an important role in the proliferation of cancer cells and the progression of a variety of tumor types^43–45^, contributing significantly to the worse outcome of patients with tumors exhibiting a high level of ALDH1A1. In addition, inhibition of the RA signaling using the pan-RAR antagonist can dramatically reduce brain metastasis from osimertinib-refractory lung cancer cells^45^. In our current study, we identified the RARE sequence in the promoter region of the *POLQ* gene and confirmed the positive regulation of the *POLQ* gene transcription by the RA signaling. Furthermore, we also demonstrated that the ALDH1A1-RA-Polθ axis plays a critical role in PARPi resistance in ovarian cancer cells.

Given that ALDH1A1 is frequently elevated in multiple cancer and contributes to cancer cell proliferation and chemoresistance via multiple mechanisms, specific inhibition of ALDH1A1 activity is considered a promising strategy for the cancer treatment. In our previous study, we have tested the potential of the ALDH1A1 inhibitor NCT-501 in sensitizing *BRCA2* mutated EOC cell lines to PARPi^12^. In this study, we further tested a second-generation ALDH1A1 inhibitor, NCT-505, in patient-derived organoids. Tumor organoids are 3D structures derived from human tumor tissues, they can anatomically and functionally mimic the tumor from which they were derived^26,46^, and can enable us to determine the therapeutic potential of the ALDH1A1 inhibitor in a more clinically relevant fashion. We clearly showed that NCT-505 can synergistically enhance the toxicity of olaparib in PDOs characterized with BRCA1/2 mutation and ALDH1A1 expression. Interestingly, we did not find synergism between olaparib and NCT-505 in killing PDOs with high genomic instability scores, although these PDOs express high levels of ALDH1A1 as well. GIS was calculated based upon aggregate analysis of Loss of Heterozygosity (LOH), Telomeric Allelic Imbalance (TAI), and Large-scale State Transitions (LST), which are not necessarily caused by HR deficiency. Thus, we need to use BRCA1/2 mutation or other HRD index and the ALDH1A1 expression status to select patients who will benefit PARPi and ALDH1A1 inhibitor combination treatment. Of course, only a limited number of patients in this study was tested, and increasing the number of patients is needed to confirm our findings and establish the patient selection criteria for PARPi and ALDH1A1 inhibitor combination treatment to achieve favorable treatment outcomes.

**Fig. 7:**
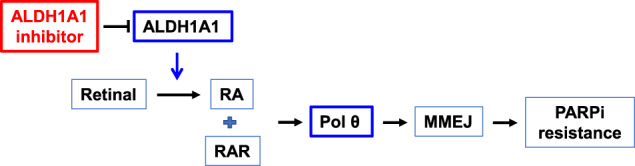
Schematic illustration of the mechanism through which ALDH1A1 promotes PARPi resistance. ALDH1A1 promotes the synthesis of RA, which binds to the RAR to activate the transcription of *POLQ*/Polθ, thereby enhancing MMEJ and leading to PARPi resistance. The ALDH1A1 inhibitor is able to block ALDH1A1-induced *POLQ*/Polθ transcription activation, and thus sensitize ovarian cancer cells to PARPi.

In summary, we demonstrated that ALDH1A1 facilitates the production of RA, which activates the transcription of *POLQ* via binding to the RAR at the RARE in the promoter region of the *POLQ* gene, thereby enhancing MMEJ and leading to PARPi resistance in HR-deficient ovarian cancer cells. Combination treatment with olaparib and the ALDH1A1 inhibitor is able to synergistically reduce tumor cell viability in the PDOs with mutated BRCA1/2 and positive ALDH1A1 expression (Fig. 7). This study not only provides a novel mechanism for PARPi resistance in ovarian cancer, but also tested the therapeutic potential of a new combination therapy in clinic relevant PDO models and suggested the patient selection criteria for this combination treatment.

## Methods

### Cell lines and reagents

Epithelial ovarian cancer (EOC) cell lines PEO1 (BRCA2^−/−^) were kindly provided by Dr. Rugang Zhang (Wistar Institute), Kuramochi (BRCA2^+/−^) and OVCAR4 (BRCA-WT) cells were kindly provided by Dr. Adam Karpf (Eppley Institute, University of Nebraska), UWB1.289 (BRCA1^−/−^) and OVCAR3 (BRCA-WT) cells were purchased from ATCC (Manassas, VA). UWB1.289 cells were cultured in a 1:1 mixed medium solution containing 50% RPMI-1640 medium and 50% MEGM with bullet kit (Lonza, MEGM bullet kit, CC-3150) supplemented with 3% fetal bovine serum (FBS). Other cells were cultured in RPMI-1640 media supplemented with 10% FBS. 100 μg/ml streptomycin and 100 units/ml of penicillin solution were added to the media. Cells were cultured at 37 °C in a humidifying chamber with 5% CO_2_. All the experiments were performed using cells within 20 passages after being revived from liquid nitrogen. All-*trans*-Retinoic Acid (ATRA) was purchased from Sigma Aldrich (Cat. No. R2625), RAR agonist CH55 (Cat. No. 2020) and RAR antagonist AGN 193109 (Cat. No. 5758) were purchased from Tocris Biosciences. Olaparib was purchased from MedChemExpress (Cat. No. HY-10162). The ALDH1A1 inhibitor NCT-505 was synthesized by the National Center for Advancing Translational Sciences (NCATS). All the above-mentioned drugs were dissolved using DMSO and fresh stock solutions were made each time before use.

### Plasmid, siRNA and transfection

pcDNA3-HA-ADH (ALDH1A1) plasmid was a gift from Steven Johnson (Addgene plasmid # 11610), pcDNA3.1-C-(k)DYK-CYP26A1 was purchased from GenScript (Piscataway, NJ). ON-TARGETplus SMARTPool Human ALDH1A1 siRNA (Cat. ID: L-008722), ON-TARGETplus SMARTPool Human POLQ siRNA (L-015180-), ON-TARGETplus Human siRARA-1 (5’-GACAAGAACUGCAUCAUCA-3’), siRARA-4 (5’-GAGCAGCAGUUCUGAAGAG-3’), and non-targeting control siRNA (5′-UUCUCCGAACGUGUCACGU-3′) were purchased from Horizon (Lafayette, CO). MISSION shALDH1A1-1 (5’-GCTGATTTAATCGAAAGAGAT-3’), shALDH1A1-4 (5’-GCCAAATCATTCCTTGGAATT-3’) were purchased from Millipore Sigma (Burlington, MA). Plasmids (0.5 μg/ml) and siRNA (100 nM) were transfected into cells using either electroporation (NEPA-21 Electroporator, Bulldog Bio, Portsmouth, NH), or Lipofectamine 2000 (Thermo Fisher Scientific, Waltham, MA), according to the manufacturer’s instruction.

### RNA sequencing and data analysis

PEO1 cells were transfected with ALDH1A1 siRNA or control siRNA for 48 h. Total RNA was isolated using Norgen Total RNA Purification Kit (Norgen Biotek) following manufacturer’s instructions and subjected to RNA sequencing (RNA-seq) as described previously^47^. Briefly, after the quality control procedure, mRNA from samples were enriched using oligo(dT) beads, then fragmented randomly in fragmentation buffer, followed by cDNA synthesis using random hexamers and reverse transcriptase. cDNA library was then constructed and subjected to sequencing by Novogene. The original raw data from Illumina HiSeq were transformed to Sequenced Reads by base calling, raw reads were filtered to remove reads containing adapters or reads of low quality, and mapped to a reference genome using TopHat2 algorithm. Gene expression levels were measured by transcript abundance, and expressed as number of Fragments Per Kilobase of transcript sequence per Millions (FPKM). The differential gene expression analysis was conducted using DESeq^48^.

### Cell viability assay

Cells were seeded in a 96-well plate (200–1000 cells/well) and incubated for 24 h. Cells were then treated with different concentrations of olaparib for 7 days. Cells were washed with PBS thrice and fixed using 3.7% formaldehyde solution for 30 min. Later, the plates were stained using 1% methylene blue for 60 min. Plates were rinsed with running tap water and allowed to dry. Overall, 100 μl of dissolving solution (10% acetic acid, 50% methanol in water) was added to each well and absorbances were recorded using spectrophotometer at 609 nm. The viability of vehicle-treated cells were set to 100% to calculate the relative cell viability of treated cells.

### Cell lysate preparation and Immunoblotting

Cells were harvested at required time point. Whole-cell lysates were prepared by adding protein lysis buffer [2% SDS, 10% glycerol, 62 mM TRIS-HCL pH 6.8, and a complete miniprotease inhibitor mixture (Roche Applied Sciences, Branford, CT)] and boiled for 10 min. Protein was quantified using the DC protein assay kit (Bio-Rad, Hercules, CA). Equal amount of protein was loaded in either 8-16% pre-cast gel (Thermo Fisher Scientific) or 4-15% pre-cast gel (BIO-RAD, Hercules, CA) for electrophoresis, and transferred to nitrocellulose membranes. Protein bands were immuno-detected using the following antibodies: mouse anti-Polθ antibody (kindly provided by Dr. Jean-Sebastien Hoffmann, Cancer Research Center of Toulouse, France)^49^, rabbit anti-ALDH1A1 antibody (Cell Signaling Technologies, Cat. No. 54135, 1:1000), rabbit anti-RARα antibody (Diagenode, Cat. No. C15310155, 1:200), rabbit anti-Polθ antibody (Affinity Biosciences, Cat. No. DF13563, 1:1000), mouse anti-GAPDH antibody (Santa Cruz Biotechnology, Cat. No. sc-365062, 1: 3000). Protein bands were visualized with ECL substrates and CCD imaging system. All blots derive from the same experiment and that they were processed in parallel. The density of each band was measured using Image J, the relative amounts of analyzed proteins were further calculated by normalizing to the corresponding loading control, then to the experimental control in each group, which is set to 1. All uncropped blots were included in Supplementary data (Supplementary Fig. 5).

### RNA Isolation and quantitative real-time PCR (qRT-PCR)

Total RNA was extracted from cancer cells using TRIZOL reagent (Thermo Fisher Scientific). In total, 1 μg of RNA was used to synthesize cDNA using the Promega Reverse Transcription System (Cat. No. A3500, Promega, Madison, WI) in a 20 μl reaction. After diluted four times, 2.5 μl of cDNA was amplified by Fast SYBR Green Master Mix (Applied Biosystems, Cat. No. 43858610) in a 20 μl reaction. Reactions were run on the QuantStudio 3 Real-Time PCR system (Applied Biosystems). The primers used for PCR are listed as follows: *ALDH1A1*, forward: 5’-TGTTAGCTGATGCCGACTTG-3’, reverse: 5’-TTCTTAGCCCGCTCAACACT-3’; *GAPDH*, forward: 5’-GAAGGTGAAGGTCGGAGT-3’, reverse: 5′- GAAGATGGTGATGGGATTTC -3′; *POLQ*, forward: 5’-TATCTGCTGGAACTTTTGCTG-3’; reverse: 5’-CTCACACCATTTCTTTGATGGA-3’; *CYP26A1*, forward: 5’-GCTGCCTCTCTAACCTGCAC-3’; reverse: 5’-TGCTTTAGTGCCTGCATGT-3’; *RARB*, forward: 5’-CTACACTGCGAGTCCGTCTT-3’, reverse: 5’-CAGAGCTGGTGCTCTGTGTT-3’; *RARA*, forward: 5’-ATCGAGACCCAGAGCAGCAG-3’, reverse: 5′- CCTGGTGCGCTTTGCGAACC-3′.

### Immunofluorescence

Cells were seeded on a cover slip in a 35 mm dish (1 × 10^4^) and cultured for 24 h. Cells were treated with either DMSO or olaparib (10 μM) for 1 h and further cultured in drug-free media for another 1 h or 12 h. Cells were washed with cold PBS, fixed and permeabilized with 2% paraformaldehyde and 0.5% Triton-X-100 at 4 °C for 30 min. Cells were blocked with 20% normal goat serum, incubated with the mouse anti-γH2AX antibody (Cat. No. 05-636, Millipore Sigma, 1:200) for overnight. After washing 4 times with PBST (PBS containing 0.1% Tween-20), cells were further incubated with the goat anti-mouse IgG conjugated with Alexa Fluor 488 (Thermo Fisher Scientific, 1:200) at room temperature for 1 h. After washed with PBST for 4 times, cells were mounted in an antifade containing medium with 0.75 mg/ml of DAPI (Vector Laboratories, Burlingame, CA) as a DNA counter stain. Cell images were visualized using the Revolve fluorescent microscope (ECHO, San Diego, CA).

### Chromatin immunoprecipitation (ChIP)

After treatment, cells were fixed with 1% formaldehyde for 10 min at room temperature. 120 mM glycine was then added to neutralize fixation. Cells were washed with PBS and harvested using cell scrapper. Cell pellets were collected by centrifuging the tube at 2500 rpm for 10 min at 4 °C. The ChIP-IT Express Enzymatic kit (Cat. No. 53009, Active Motif, Carlsbad, CA) was used to conduct ChIP according to the manufacturer’s instruction. The following antibodies were used for immunoprecipitation: rabbit anti-RARα antibody (C15310155, Diagenode), rabbit anti-H3K27ac antibody (Cat. No. 9649, Cell Signaling Technologies), rabbit anti-H3K27me3 (Cat. No. 39155, Active Motif), and normal rabbit IgG (Cat. No. 2729, Cell Signaling Technologies). Immunoprecipitated DNA was purified by Phenol/chloroform extraction and quantified by qPCR analysis with the primer set corresponding to the specific region of the *POLQ* gene promoter: forward: 5’-CCTGACCTGAAGAGGCTGTG-3’, reverse: 5’-TGGTTCGAATGCTATTTTCCAGA-3’.

### Patient-derived organoid (PDO) culture

HGSOC tumor tissues were obtained from the Tissue Procurement Service (TPS) at the Ohio State University within 4 hours after surgery in accordance with a protocol approved by the Ohio State University’s IRB (2017C0033). Written informed consent was obtained from all human donors for the use of their tissues. Organoid cultures were established from these tumor tissues as described previously^50^. Organoids were seeded at 1 × 10^4^ cells/well in 75% Matrigel in a 96-well plate and treated with different concentrations of olaparib and NCT-505 separately or in combination for 7 days. Cell viability was determined using the CellTiter-Glo 3D Cell Viability Assay (Promega, Cat. No. G7571). BRCA1/2 mutation status and genomic instability score (GIS) were obtained from the Department of Pathology. Genomic instability score (GIS) was calculated based on aggregate analysis of Loss of Heterozygosity (LOH), Telomeric Allelic Imbalance (TAI), and Large-scale State Transitions (LST). GIS ≥ 42 was considered positive.

### Immunohistochemistry (IHC)

Formalin-fixed paraffin-embedded (FFPE) tissue sections were subjected to deparaffinization and rehydration. Endogenous peroxidase was quenched by 3% hydrogen peroxide. The epitope retrieval was performed using Target Retrieval Solution (Dako S1699, Agilent Technologies, Santa Clara, CA) for 27 min at 96 °C in a vegetable steamer. Slides were incubated with primary rabbit antibody against ALDH1A1 (Cat. No. A700-089, Bethyl Laboratories, Montgomery, TX, 1:100) for overnight at 4 °C, then with biotinylated goat anti-rabbit antibody (Vector BA-1000, 1:200) for 30 min at room temperature. The VectaStain Elite Standard ABC Kit-HRP (Vector PK6100) was used to detect the biotinylated antibodies. Finally, slides were stained with Dako DAB+ Chromogen, and counterstained with Dako Flex Hematoxylin. All immunostains were reviewed by at least two pathologists. The IHC results were categorized based on the percentage of immunoreactive tumor cells as the following: negative (<1%), low positive (1–9%), intermediate positive (≥10%, but <50%), or high positive (≥ 50%). We used a cut off value of 1% for ALDH1A1 positivity in current study.

### Statistical analysis

Descriptive statistics, i.e., means ± standard deviation (SD), are shown in the figures. Two sample *t* tests or ANOVA were performed for comparing two groups or more than two groups, respectively. Linear mixed-effect models were used to analyze the combination effect of olaparib and NCT-505 on cell viability. *P* < 0.05 was considered statistically significant. All tests were two-sided.

### Reporting summary

Further information on research design is available in the Nature Research Reporting Summary linked to this article.

## Supplementary information

REPORTING SUMMARY

Supplementary Figures

## Data Availability

The data generated in this study are available within the article and its supplementary data files. RNA sequencing data is freely available within the NCBI GEO database (GSE82304, GSE226018). Data generated in this study are also available from the corresponding authors upon reasonable request.
