# Supplementary material for: ALDH1A1 promotes PARP inhibitor resistance by enhancing retinoic acid receptor-mediated DNA polymerase θ expression
Source: NPJ Precis Oncol. 2023 Jul 10;7:66. doi: 10.1038/s41698-023-00411-x (PMC10333219; doi:10.1038/s41698-023-00411-x)
Supplement: Supplementary file 1 — REPORTING SUMMARY [file 41698_2023_411_MOESM1_ESM.pdf]

nature portfolio

Corresponding author(s): Qi-En Wang

Last updated by author(s): Mar 2, 2023

Reporting Summary

Nature Portfolio wishes to improve the reproducibility of the work that we publish. This form provides structure for consistency and transparency in reporting. For further information on Nature Portfolio policies, see our Editorial Policies and the Editorial Policy Checklist.

Statistics

For all statistical analyses, confirm that the following items are present in the figure legend, table legend, main text, or Methods section.

n/a	Confirmed	
☐	☒	The exact sample size (*n*) for each experimental group/condition, given as a discrete number and unit of measurement
☐	☒	A statement on whether measurements were taken from distinct samples or whether the same sample was measured repeatedly
☐	☒	The statistical test(s) used AND whether they are one- or two-sided<br>*Only common tests should be described solely by name; describe more complex techniques in the Methods section.*
☒	☐	A description of all covariates tested
☒	☐	A description of any assumptions or corrections, such as tests of normality and adjustment for multiple comparisons
☐	☒	A full description of the statistical parameters including central tendency (e.g. means) or other basic estimates (e.g. regression coefficient) AND variation (e.g. standard deviation) or associated estimates of uncertainty (e.g. confidence intervals)
☐	☒	For null hypothesis testing, the test statistic (e.g. *F*, *t*, *r*) with confidence intervals, effect sizes, degrees of freedom and *P* value noted<br>*Give P values as exact values whenever suitable.*
☒	☐	For Bayesian analysis, information on the choice of priors and Markov chain Monte Carlo settings
☒	☐	For hierarchical and complex designs, identification of the appropriate level for tests and full reporting of outcomes
☒	☐	Estimates of effect sizes (e.g. Cohen's *d*, Pearson's *r*), indicating how they were calculated

Our web collection on statistics for biologists contains articles on many of the points above.

Software and code

Policy information about availability of computer code

Data collection	N/A
Data analysis	N/A

For manuscripts utilizing custom algorithms or software that are central to the research but not yet described in published literature, software must be made available to editors and reviewers. We strongly encourage code deposition in a community repository (e.g. GitHub). See the Nature Portfolio guidelines for submitting code & software for further information.

Data

Policy information about availability of data

All manuscripts must include a data availability statement. This statement should provide the following information, where applicable:

- Accession codes, unique identifiers, or web links for publicly available datasets
- A description of any restrictions on data availability
- For clinical datasets or third party data, please ensure that the statement adheres to our policy

The data generated in this study are available within the article and its supplementary data files. RNA sequencing data is freely available within the NCBI GEO database (GSE82304, GSE226018). Data generated in this study are also available from the corresponding authors upon reasonable request.

Human research participants

Policy information about studies involving human research participants and Sex and Gender in Research.

Reporting on sex and gender	N/A
Population characteristics	N/A
Recruitment	N/A
Ethics oversight	N/A

Note that full information on the approval of the study protocol must also be provided in the manuscript.

Field-specific reporting

Please select the one below that is the best fit for your research. If you are not sure, read the appropriate sections before making your selection.

☐ Life sciences ☐ Behavioural & social sciences ☐ Ecological, evolutionary & environmental sciences

For a reference copy of the document with all sections, see nature.com/documents/nr-reporting-summary-flat.pdf

Life sciences study design

All studies must disclose on these points even when the disclosure is negative.

Sample size	N/A
Data exclusions	N/A
Replication	N/A
Randomization	N/A
Blinding	N/A

Behavioural & social sciences study design

All studies must disclose on these points even when the disclosure is negative.

Study description	N/A
Research sample	N/A
Sampling strategy	N/A
Data collection	N/A
Timing	N/A
Data exclusions	N/A
Non-participation	N/A
Randomization	N/A

Ecological, evolutionary & environmental sciences study design

All studies must disclose on these points even when the disclosure is negative.

Study description	N/A
Research sample	N/A
Sampling strategy	N/A
Data collection	N/A
Timing and spatial scale	N/A
Data exclusions	N/A
Reproducibility	N/A
Randomization	N/A
Blinding	N/A

Did the study involve field work? ☐ Yes ☒ No

Reporting for specific materials, systems and methods

We require information from authors about some types of materials, experimental systems and methods used in many studies. Here, indicate whether each material, system or method listed is relevant to your study. If you are not sure if a list item applies to your research, read the appropriate section before selecting a response.

Materials & experimental systems

n/a	Involved in the study	
☐	☒	Antibodies
☐	☒	Eukaryotic cell lines
☒	☐	Palaeontology and archaeology
☒	☐	Animals and other organisms
☒	☐	Clinical data
☒	☐	Dual use research of concern

Methods

n/a	Involved in the study	
☒	☐	ChIP-seq
☒	☐	Flow cytometry
☒	☐	MRI-based neuroimaging

Antibodies

Antibodies used

Mouse anti-Pol θ antibody, provided by Dr. Jean-Sebastien Hoffmann, Cancer Research Center of Toulouse, France
Rabbit anti-Pol θ antibody, Affinity Biosciences, DF13563
Rabbit anti-ALDH1A1 antibody, Cell Signaling Technologies, 54135
Rabbit anti-RARα antibody, Diagenode, C15310155
Mouse anti-GAPDH antibody, Santa Cruz Biotechnology, sc-365062
Mouse anti-γH2AX antibody, MilliporeSigma, 05-636
Rabbit anti-H3K27ac antibody, Cell Signaling Technologies, 9649
Rabbit anti-H3K27me3 antibody, Active Motif, 39155

Validation

All primary antibodies used in this study were validated by the manufacture. Validate data / citations can be found on the manufacture website by searching the antibody catalogue number provided in the manuscript.

Eukaryotic cell lines

Policy information about cell lines and Sex and Gender in Research

Cell line source(s)

PEO1: provided by Dr. Rugang Zhang (Wistar Institute)
Kuramochi: provided by Dr. Adam Karpf (Eppley Institute, University of Nebraska)
OVCAR4: provided by Dr. Adam Karpf (Eppley Institute, University of Nebraska)
UWB1.289: Purchased from ATCC

	OVCAR3: Purchased from ATCC
Authentication	All cell lines were authenticated using the DNA (short tandem repeat) profiling at ATCC and OSUCCC Genomics Shared Resource.
Mycoplasma contamination	All cell line were tested negative for mycoplasma contamination.
Commonly misidentified lines (See ICLAC register)	N/A
