# Supplementary material for: ALDH1A1 promotes PARP inhibitor resistance by enhancing retinoic acid receptor-mediated DNA polymerase θ expression
Source: NPJ Precis Oncol. 2023 Jul 10;7:66. doi: 10.1038/s41698-023-00411-x (PMC10333219; doi:10.1038/s41698-023-00411-x)
Supplement: Supplementary file 2 — Supplementary Figures [file 41698_2023_411_MOESM2_ESM.pdf]

ALDH1A1 promotes PARP inhibitor resistance by enhancing retinoic acid receptor-mediated DNA polymerase θ expression

Lavudi K, et al.

Supplementary Information

Supplementary Figure 1

A

Gene	SKOV3 ALDH+ vs ALDH- Log FC	SKOV3 ALDH+ vs ALDH- P value	PEO1 siALDH1A1 vs siCtrl Log FC	PEO1 siALDH1A1 vs siCtrl P value
POLQ	0.829	0.00288	-0.5518	0.0154
XRCC1	0.386	0.0842	-0.24445	0.16165
LIG3	0.243	0.495	-0.31172	0.034842
FEN1	0.306	0.0657	-0.25349	0.084858
PARP1	-0.57	0.00559	-0.33277	0.006588
MRE11	-0.124	0.48	-0.08035	0.734519
NBN	0.135	0.582	-1.3886	3.19E-11

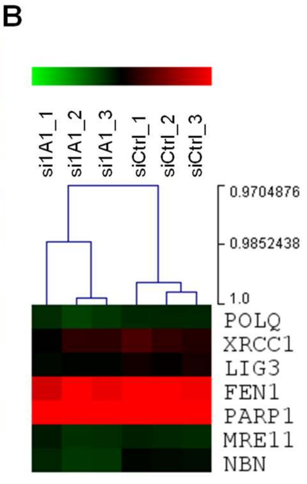

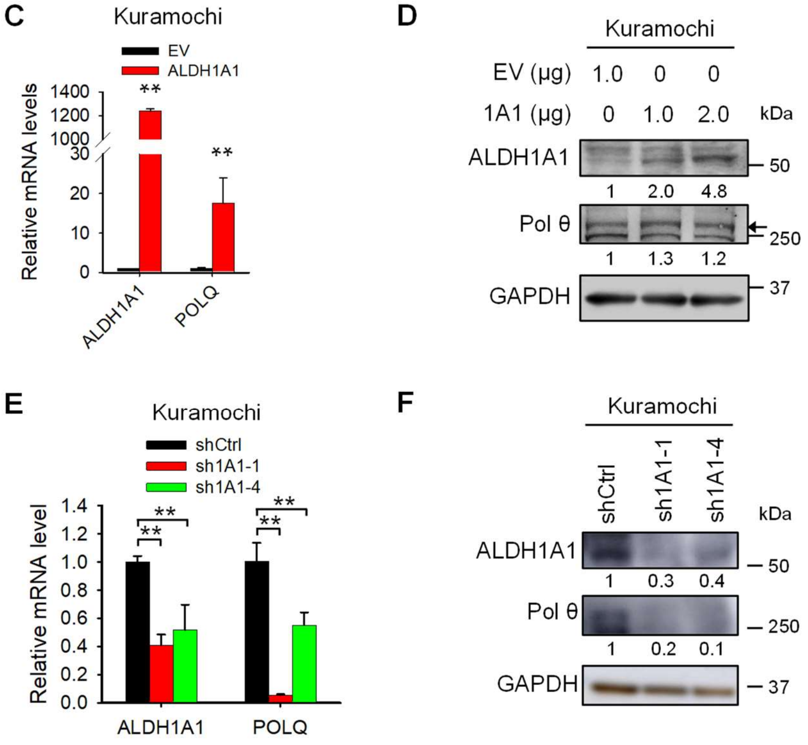

Supplemental Figure 1. ALDH1A1 increases POLQ expression. A. The differential expression of MMEJ-related genes in ALDH+ vs ALDH- cells isolated from the SKOV3 cell line, and siALDH1A1 vs siCtrl transfected PEO1 cells. **B.** Heat map of differentially expressed MMEJ-related genes in siALDH1A1 and siCtrl transfected PEO1 cells. **C.** Kuramochi cells were transfected with ALDH1A1 expression plasmids for 48 h, the mRNA level of ALDH1A1 and POLQ were determined using the qRT-PCR. **D.** Kuramochi and UWB1.289 cells were transfected with ALDH1A1 expression plasmids for 48 h, the protein levels of ALDH1A1 and Pol θ were determined using immunoblotting. GAPDH was examined to serve as a loading control. The arrow indicates the specific Pol θ band. **E, F.** Kuramochi cells were transfected with ALDH1A1 shRNA for 48 h, the mRNA and protein levels of ALDH1A1 and POLQ were determined using qRT-PCR and immunoblotting, respectively. N = 3, bar: s.d., **: $P < 0.01$. The relative amounts of analyzed proteins were listed under the corresponding band.

Supplementary Figure 2

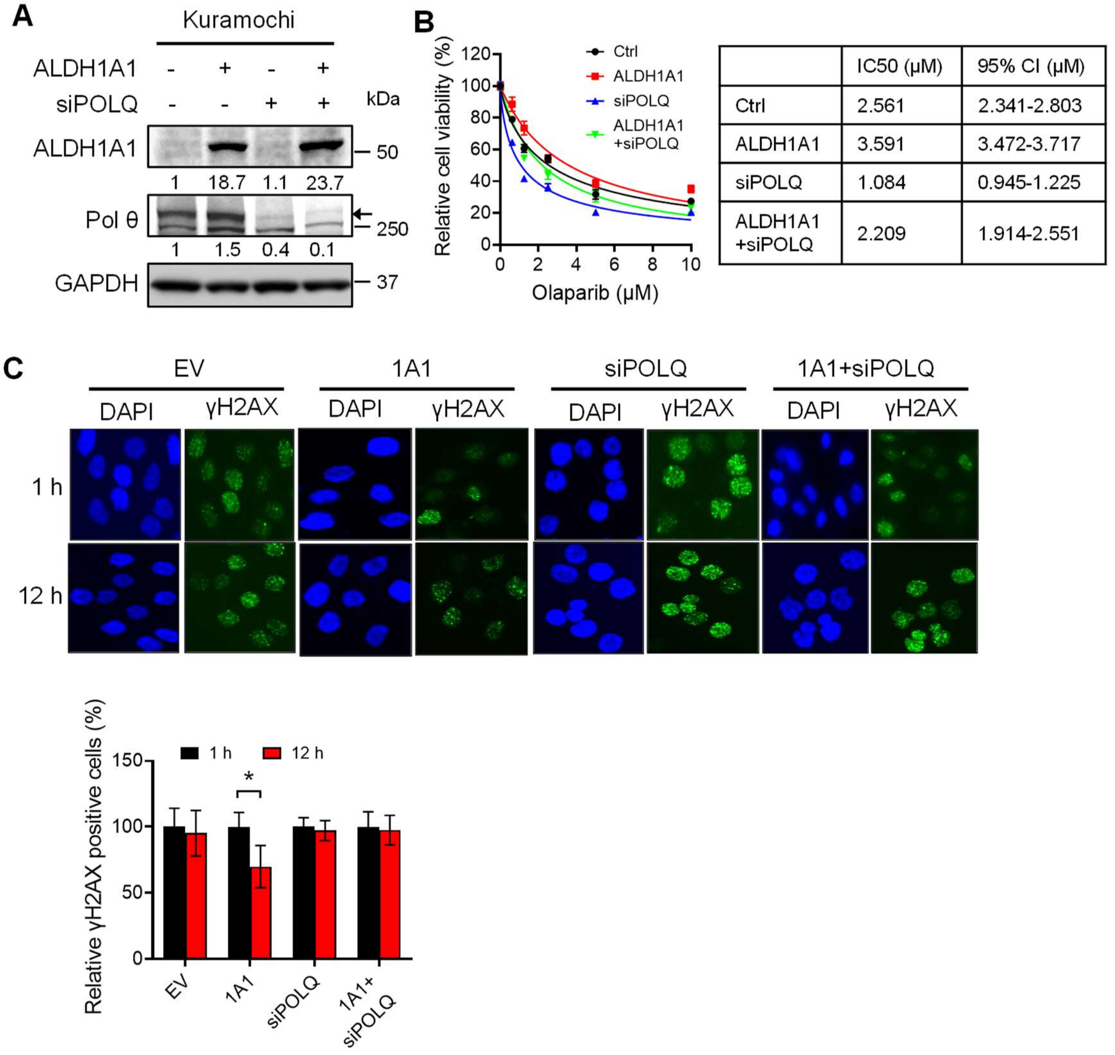

	IC50 (μM)	95% CI (μM)
Ctrl	2.561	2.341-2.803
ALDH1A1	3.591	3.472-3.717
siPOLQ	1.084	0.945-1.225
ALDH1A1 +siPOLQ	2.209	1.914-2.551

Supplemental Figure 2. ALDH1A1 enhances DNA repair and PARPi resistance via upregulating POLQ. Kuramochi cells were transfected with ALDH1A1 expression plasmids or/and siPOLQ for 48 h. The expression of ALDH1A1 and Pol θ was determined using immunoblotting (A). These cells were treated with olaparib at various doses for 7 days, the cell viability and IC50 were determined using the methylene blue assay (B). Cells were treated with olaparib (10 μM) for 1 h, and further cultured in the drug-free medium for 1 and 12 h. γH2AX foci were visualized using immunofluorescence. The percentage of γH2AX positive cells (>5 foci/cell) was calculated and normalized to 1 h time point. (C). N = 5, bar: s.d., *: $P < 0.05$. The relative amounts of analyzed proteins were listed under the corresponding band. The arrow indicates the specific Pol θ band.

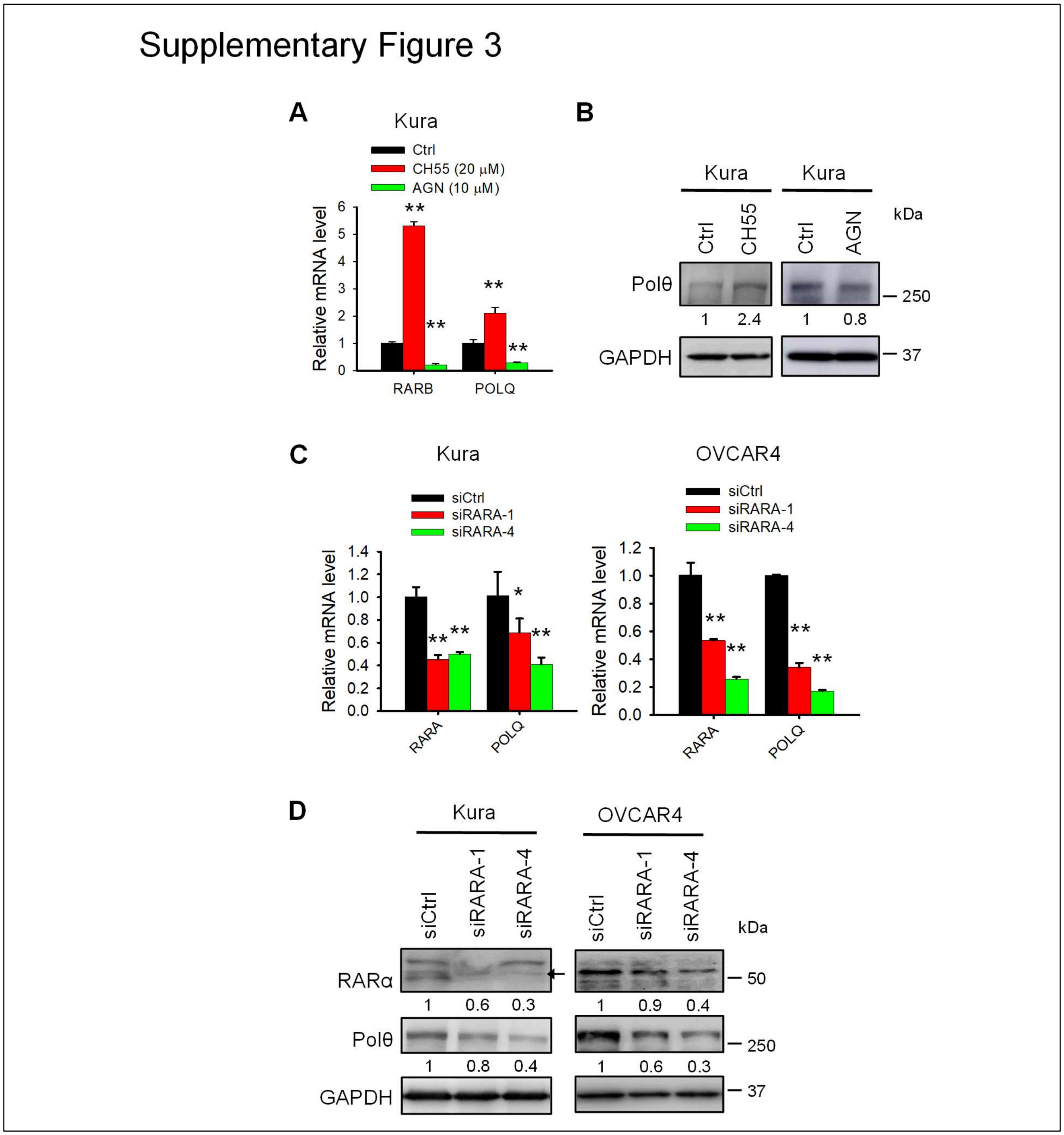

Supplemental Figure 3. POLQ is regulated by RAR signaling. A, B. Kuramochi cells were treated with RAR agonist CH55 or RAR antagonist AGN 193109 for 48 h, qRT-PCR and immunoblotting were conducted to determine the mRNA (A) and protein (B) levels of Pol θ, respectively. The mRNA level of RARB was also determined to validate the effect of RAR agonist and antagonist on the RAR signaling. **C.** Kuramochi and OVCAR4 cells were transfected with two different RARA siRNA for 48 h, qRT-PCR was conducted to determine the mRNA level of RARA and POLQ. **D.** Kuramochi and OVCAR4 cells were transfected with two different RARA siRNA for 48 h, immunoblotting was conducted to determine the protein levels of RARα and Polθ. N = 5, bar: s.d., *: $P < 0.05$. The relative amounts of analyzed proteins were listed under the corresponding band. The arrow indicates the specific RARα band.

Supplementary Figure 4

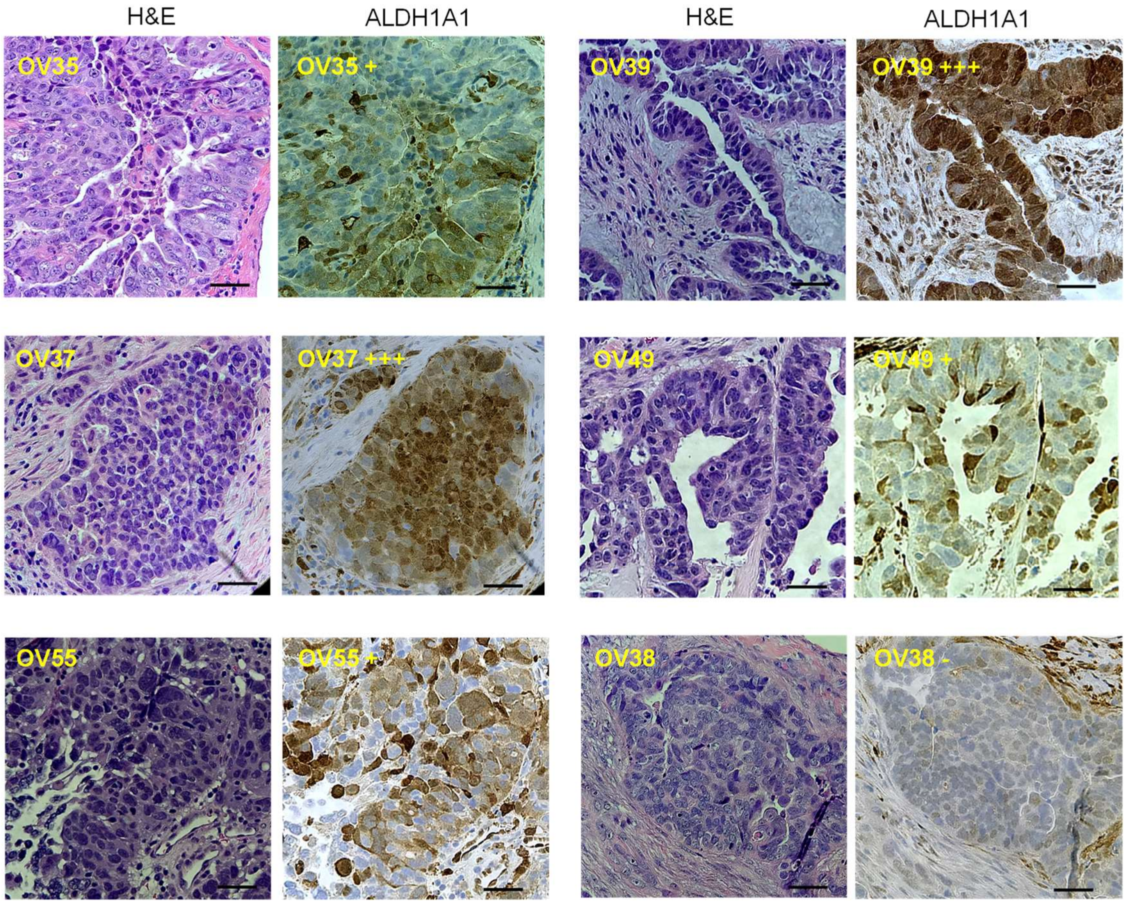

Supplemental Figure 4. H&E and IHC staining of ALDH1A1 in the HGSOC tissues. FFPE tissues were stained for H&E staining to validate the histopathology of the tumor tissues. IHC staining was performed to determine the expression status of ALDH1A1, which is semi-quantified as -, +, +++. (200X magnification, scale bar: 100 μm).

Supplementary Figure 5

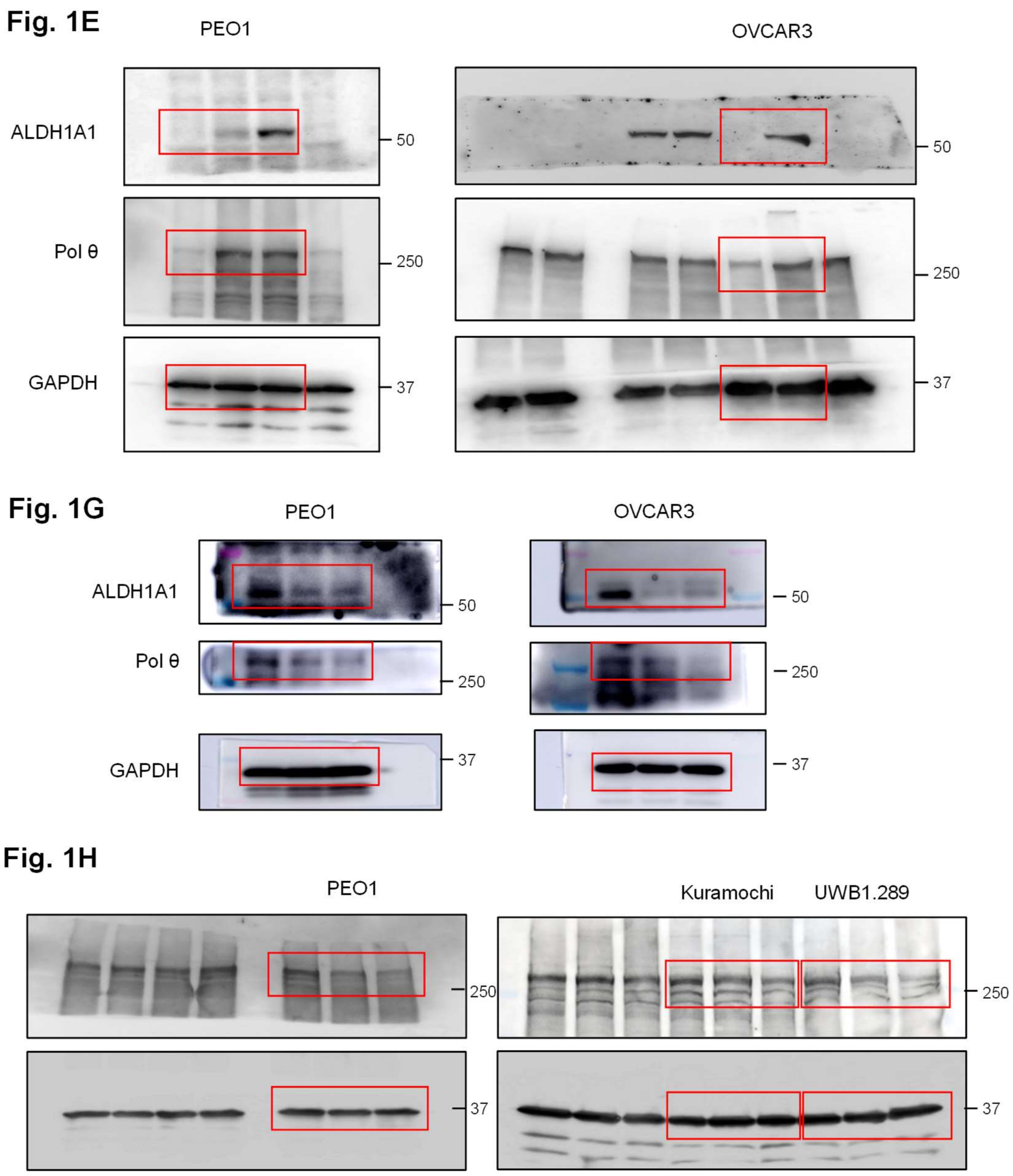

Supplementary Figure 5

Fig. 2A

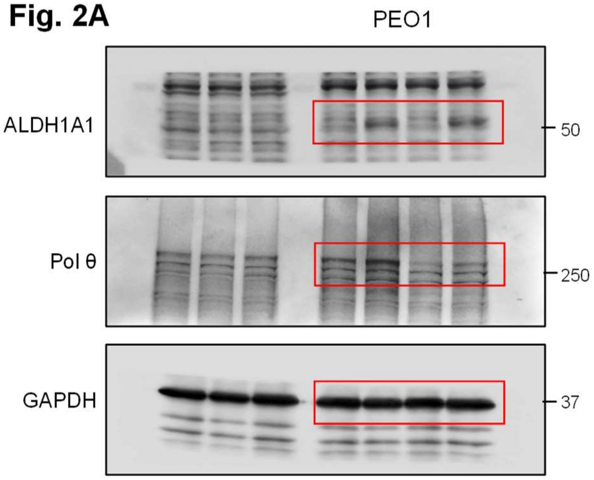

Fig. 3A

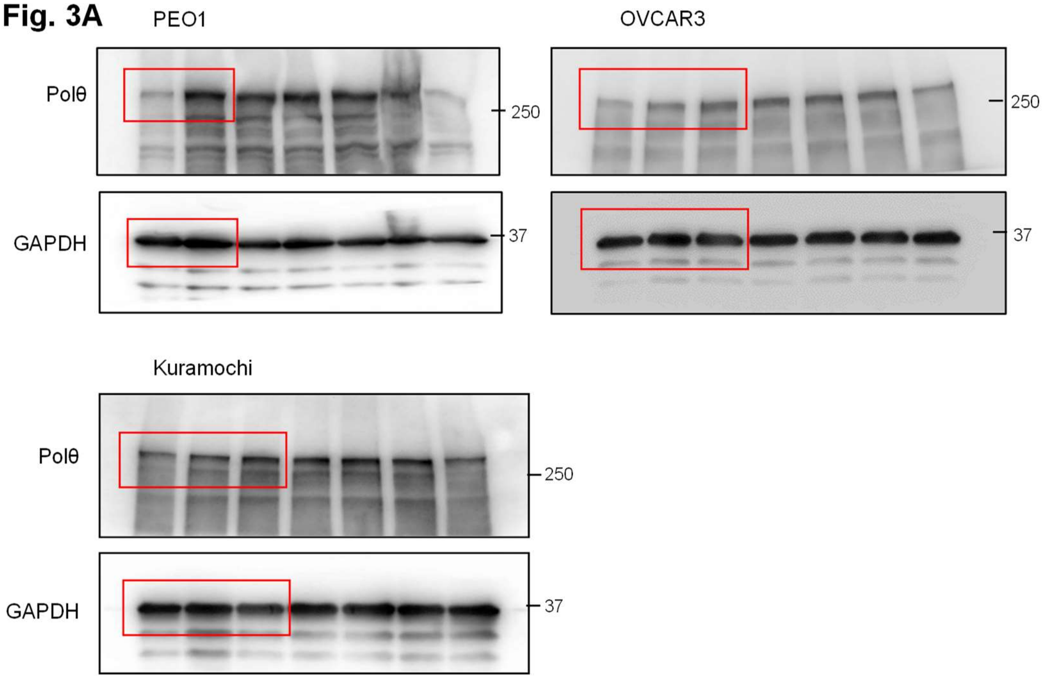

Supplementary Figure 5

Fig. 4A

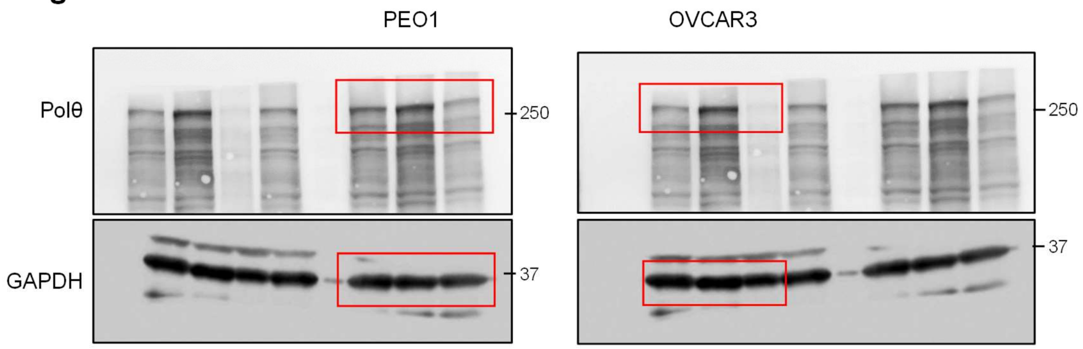

Fig. 4D

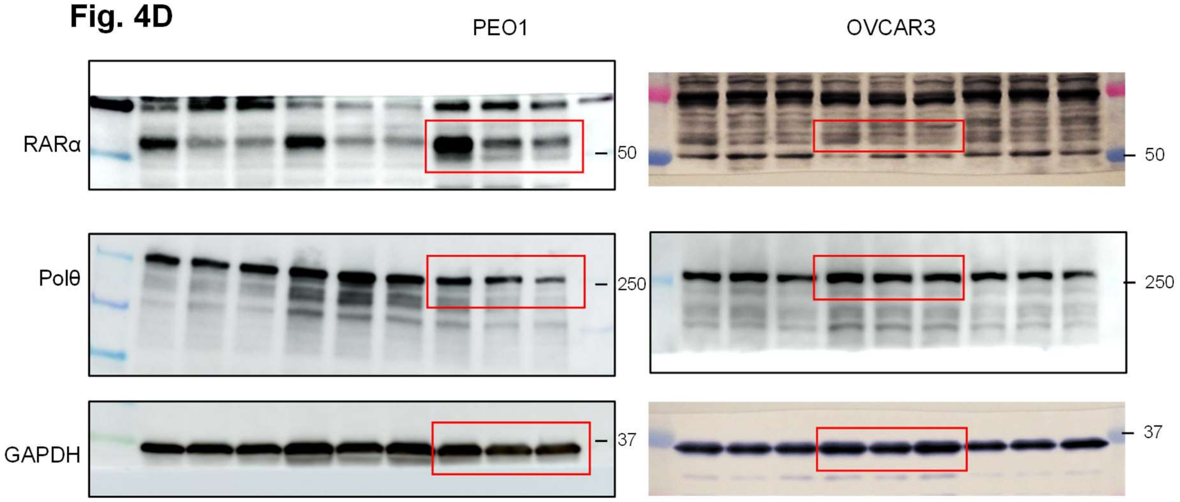

Supplementary Figure 5

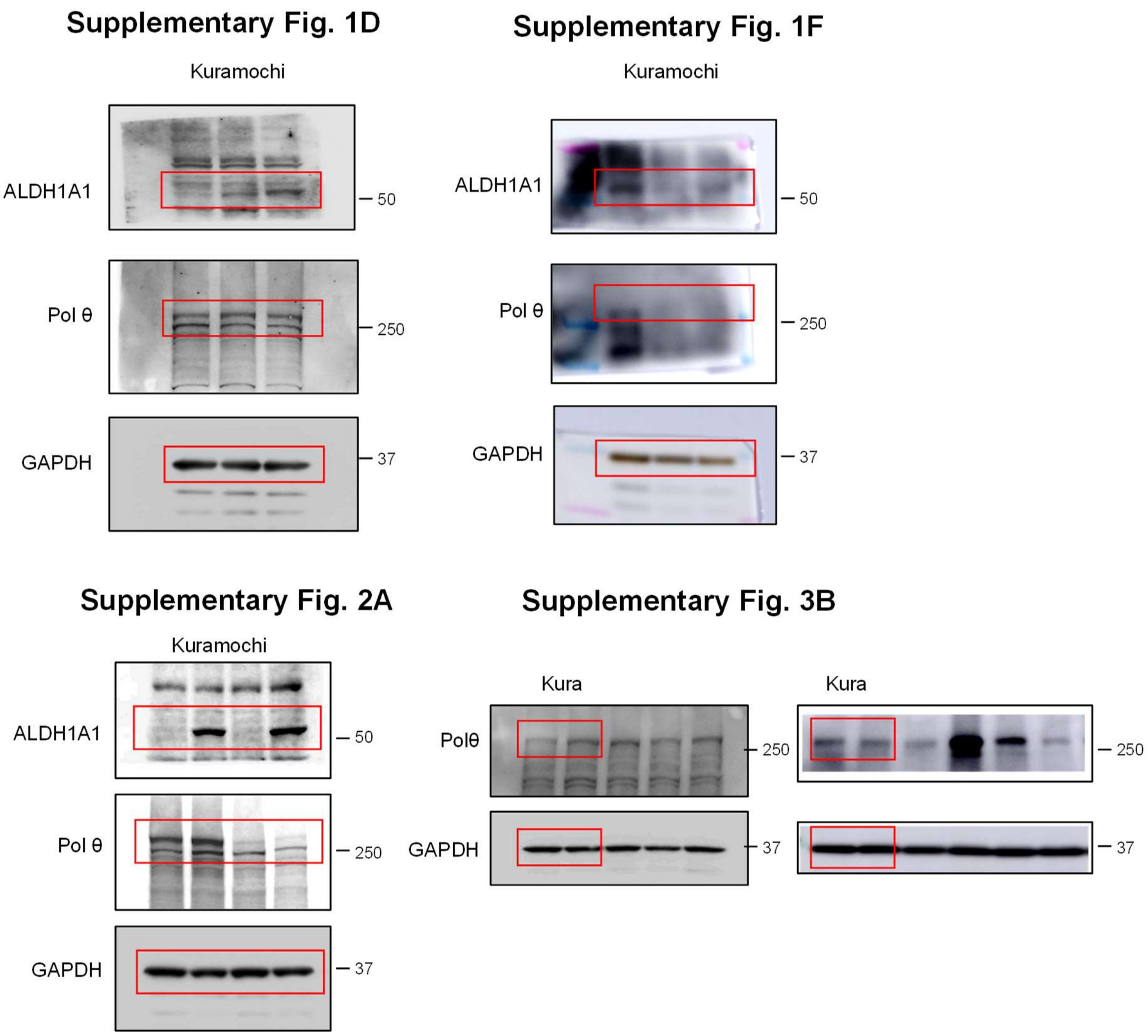

Supplementary Figure 5

Supplementary Fig. 3D

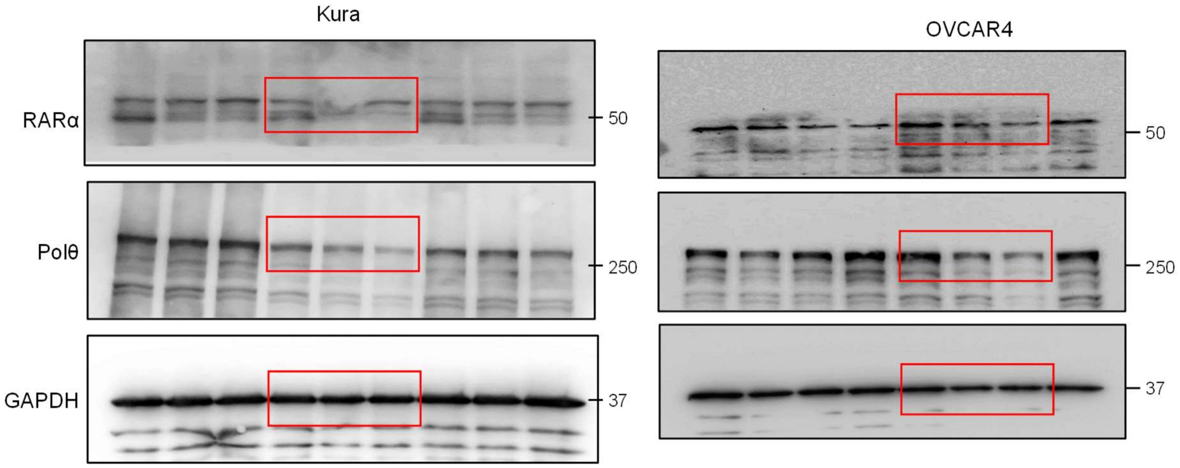

Supplemental Figure 5. Uncropped blots for main figures. Blots shown in the main article and Supplementary Information are depicted by boxed regions in each of associated uncropped scans.
